# Temperature-Controlled Molecular Bonding Hysteresis: Interphase Dynamics of a Nanoparticle-Modified Polymer Network

**DOI:** 10.1021/acs.jpclett.4c00406

**Published:** 2024-03-25

**Authors:** Andreas Klingler, Bernd Wetzel, Jan-Kristian Krüger

**Affiliations:** Leibniz-Institut für Verbundwerkstoffe, RPTU Kaiserslautern-Landau, Erwin-Schrödinger Straße 58, 67663 Kaiserslautern, Germany

## Abstract

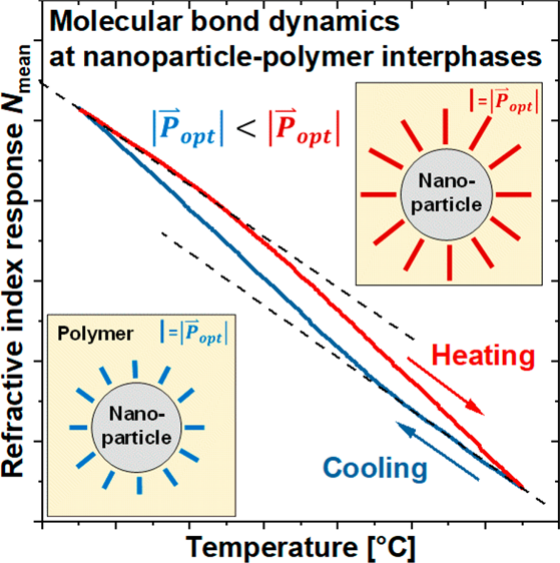

This study demonstrates the existence of temperature-induced molecular bonding hysteresis at nanoparticle–polymer interfaces in a highly cross-linked epoxy-based polymer, modified with core–shell rubber nanoparticles. This thermally induced bond hysteresis manifests itself in a hysteresis-like change of the strength of the electrical bond polarization between epoxy molecules and surface molecules of the core–shell nanoparticles. This kind of dynamic bond behavior can be controllably switched from one bond state to the other by a sufficient temperature change. The related optical remanence is evidenced by a refractive index hysteresis independent of the temperature change using the new experimental technique of temperature-modulated optical refractometry (TMOR). From the investigation of quasi-static and dynamic thermal expansion separately, TMOR allows for the conclusion that the observed hysteresis is caused by the specific refractivity and not the dipole number density.

Cross-linked polymers, such as epoxy-based thermosets (EP), are indispensable in modern applications and serve, e.g., as adhesives or coatings or are used as polymer matrices for fiber-reinforced composites.^1,2^ These materials are amorphous in the cross-linked state and have isotropic symmetry. Above the canonical glass transition temperature *T*_g_, they are viscoelastic solids, yet below *T*_g_, their cross-linked nature can cause extreme brittleness and makes them susceptible to cracking and mechanical failure.^3^ Various strategies have been developed to overcome the inherent brittleness of cross-linked polymers; among others, the introduction of appropriate second-phase nanoparticles into the polymer (nanocomposites).^4,5^ To activate such particle-induced “toughening mechanisms”, strong bonding between the constituents needs to prevail in the cured state of the polymer. Therefore, to enhance the interface strength between particulate modifiers and the surrounding matrix, core–shell-structured nanoparticles (CSR) are used that are composed of a ductile core polymer and a tailored polymer shell for the desired bond properties between the matrix and nanoparticles. The core serves as an energy-dissipating body, usually being a rubber, whereas the shell is a polymer matrix compatible polymer. During the cross-linking reaction of the resin system, the shell chemically or physically bonds to the molecular network. The interaction between the nanoparticle shell and the surrounding polymer matrix forms an interphase, which differs morphologically from that of the bulk matrix. After the curing reaction, it is this interphase that provides the load transfer from the polymer matrix to the particle core and, thus, defines, among other morphological effects in the polymer matrix, the degree of toughness of the nanoparticle-modified polymer.

The present publication addresses the question whether all or parts of the interface-induced molecular interactions in the nanocomposite can substantially change in a well-defined temperature range and, thus, can cause hysteresis effects, e.g., in optical or thermomechanical properties. Within this framework, we report a rather unexpected temperature-induced optical hysteresis behavior of nanoparticle–polymer interfaces using temperature-modulated optical refractometry (TMOR).^6,7^ The cycloaliphatic epoxy resin master batch contains about 30 wt % of 100 nm sized, core–shell structured nanoparticles (in the following denominated as CSR–resin) and was cured using cycloaliphatic anhydride and 1-methylimidazole. This corresponds to about 19 vol % of nanoparticles in the final polymer system (EP–CSR). The nanoparticle core is made of polybutadiene. The neat (non-particle modified) and cured cycloaliphatic epoxy resin will be referred to as “EP-neat”. A detailed description of the polymer, its components, the curing agent, and the manufacturing process are given in the Supporting Information.

TMOR is based on Abbe refractometry and uses a small sinusoidal temperature perturbation, enabling an assessment of the thermal volume expansion coefficient under static, dynamic, and kinetic conditions.^6,7^ Among the immediately accessible physical properties are the modulation frequency averaged refractive index *N*_mean_ = *n*_D_, the thermo-optical coefficient abs(d*N*_mean_/d*T*), and the thermomechanical coefficients β′ and β″, with β* = β′ – *i*β″. The quantity β* is the complex thermal volume expansion coefficient measured at a temperature modulation time  (*f* is the modulation frequency). Another external variable is the temperature *T*. The fundamental relationship between the refractive index *n* and the thermodynamic quantity mass density ρ provides the Lorenz–Lorentz relationship^8,9^
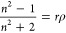
1where *r* is the so-called specific refractivity, which measures the average strength of the optical polarization.^10^ A more detailed description of TMOR and the underlying thermodynamics can be found in the Supporting Information or elsewhere.^6,7,11^

Figure 1 shows the refractive index *N*_mean_ of EP–CSR measured as a function of the temperature *T*, first in a heating and then in a cooling run between 25 and 85 °C. The heating and cooling branches form a closed cycle. The tangents of each *N*_mean_(*T*) branch at the start of the heating process (red line) as well as during the cooling process (blue line) have the same slope, reminiscent of a temperature hysteresis.

**Figure 1:**
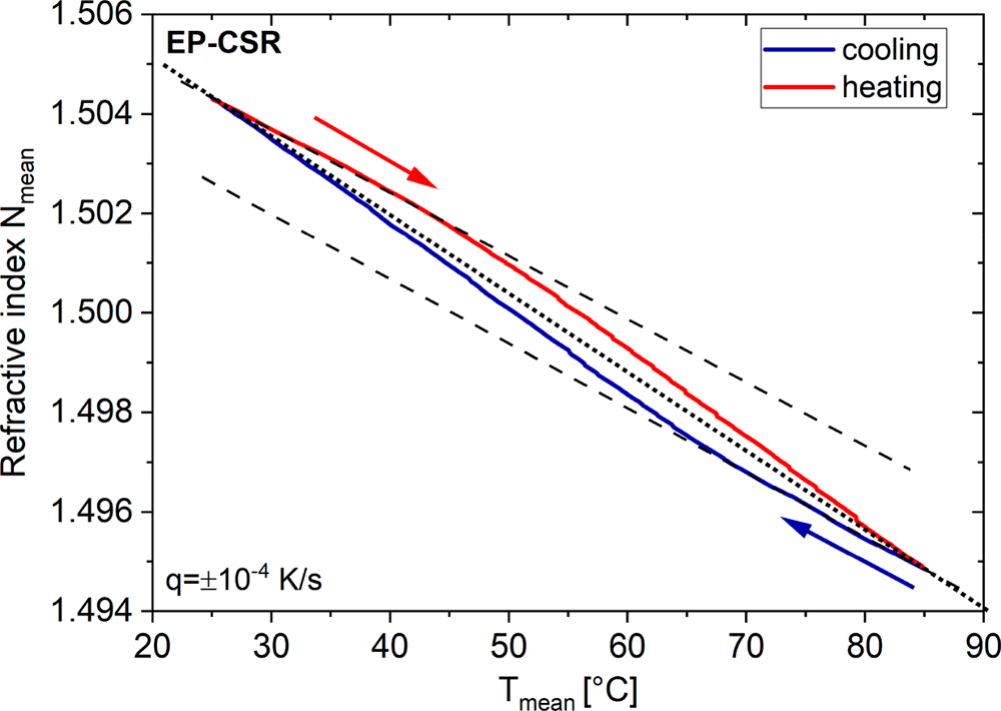
Temperature hysteresis of *N*_mean_ of the EP–CSR system, with the temperature rate *q* = ±10^–4^ K/s.

With this taken seriously, Figure 1 indicates the existence of optical remanence in the temperature closed cycle, i.e., the ability of the system to retain a certain refractive index. Consequently, two questions arise: (i) Which morphologies within the heterogeneous nanocomposite yield to such an optical remanence? (ii) Is this remanence related to anomalous thermal mass density changes during the temperature cycle, or is it rather caused by unexpected changes of the specific refractivity *r* (cf. eq 1)? The latter interpretation would mean that the observed hysteresis is not caused by an unusual change in the dipole number density (being proportional to the mass density, ρ) but related to an unexpected change in the magnitude of a large number of molecular dipole moments (*r*). As a hypothesis, the optical dipoles in question would presumably be located in the immediate vicinity of the nanoparticle surfaces.

To elucidate question i, we have investigated the refractive index and the thermal volume expansion of the neat polymerized system, EP-neat. The refractive index function *N*_mean_(*T*) of this system, given in Figure 2, does not show any kind of anomalous behavior in the course of the cooling experiment. It shows a quasi-perfect, linear relationship between the refractive index *N*_mean_ and the temperature *T*, representing equilibrium conditions of EP-neat in this temperature interval.

**Figure 2:**
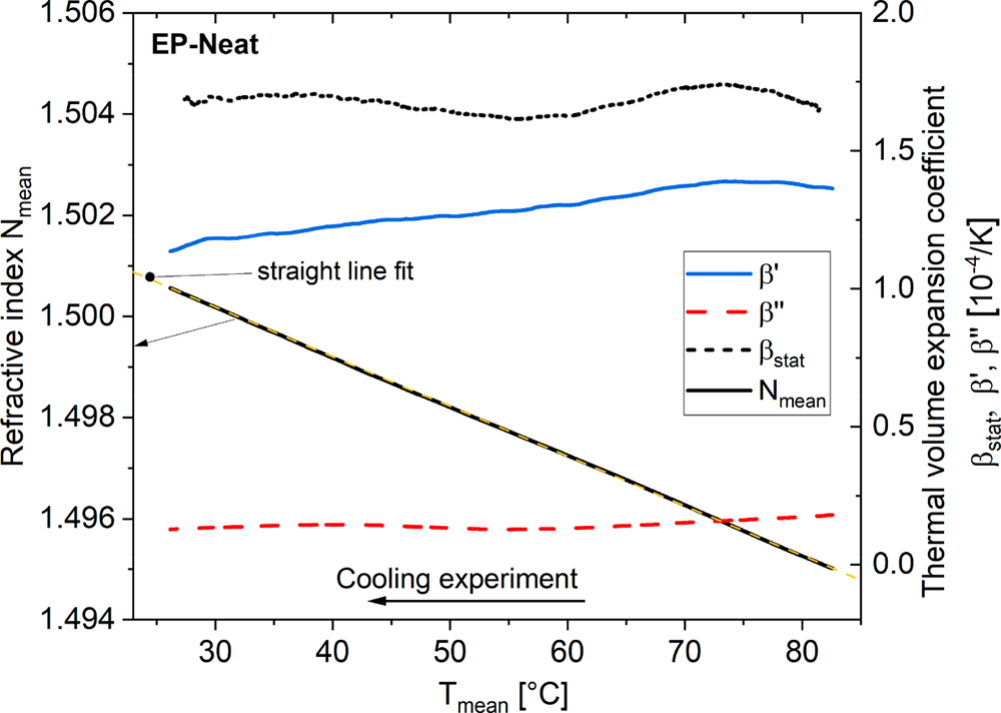
Left axis, temperature-dependent refractive index *N*_mean_(*T*) of EP-neat; right axis, thermal volume expansion coefficients β′, β″, and β_stat_ of EP-neat, measuring parameters *A*_T_ = 0.3 K, τ = 60 s, and *q* = −10^–3^ K/s. The dashed yellow straight line fit illustrates the linearity of *N*_mean_(*T*).

Moreover, the dynamic thermal volume expansion coefficient β′(*T*), measured at a probe frequency of 17 mHz, is typical for a polymer glass [β′(*T* < *T*_g_) < 2 × 10^–4^ K^–1^].^12,13^ The related loss factor β″(*T*) is unspectacularly low and in accordance with the glassy state. However, despite the low probe frequency of 17 mHz, the real part β′(*T*) of the dynamic thermal volume expansion coefficient does not coincide with the quasi-static function β_stat_(*T*). It is slightly displaced to lower values. If this is a hint to a weak secondary relaxation process in the glassy state of EP-neat, then it is still open for discussion.

From these results, it becomes clear that the observed optical remanence of the EP–CSR system, as shown in Figure 1, must be related to the presence of the CSR nanoparticles. At this point, the question remains whether the isolated CSR nanoparticles are the origin of the optical remanence or if their interaction with the polymer matrix in the polymerized state is responsible for this behavior.

To answer this, we have performed TMOR measurements of the non-reactive, non-cured, nanoparticle-filled resin (CSR–resin; Figure 3). It is assumed that the CSR nanoparticles can move freely in the surrounding liquid resin.

**Figure 3:**
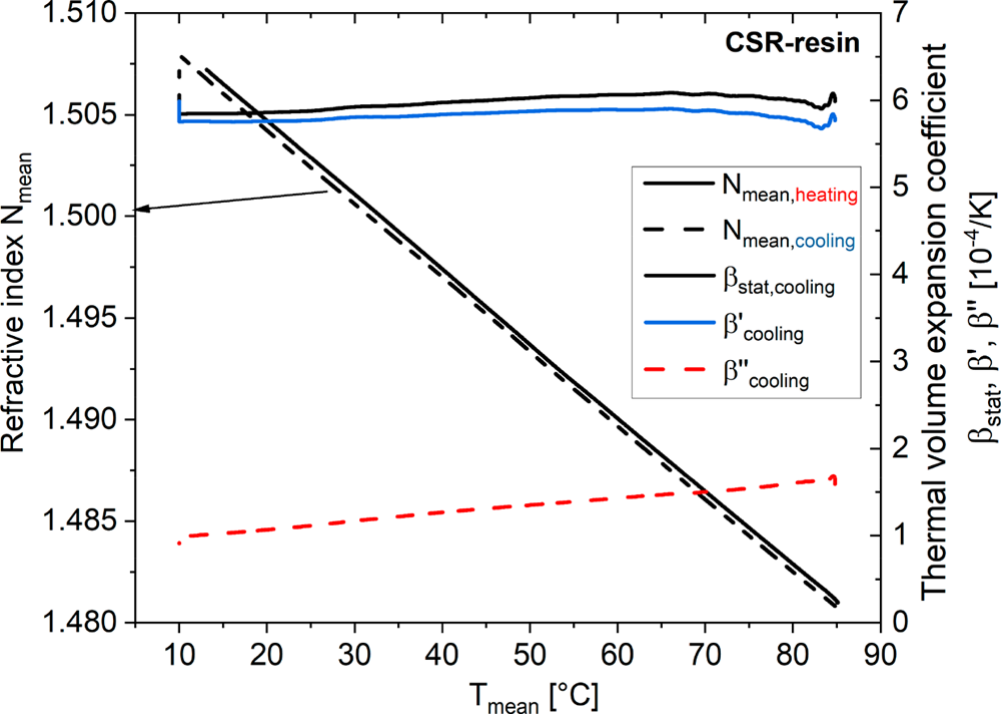
Temperature-dependent refractive index *N*_mean_(*T*) (left axis) and thermal volume expansion coefficients β′, β″, and β_stat_ (right axis) of the liquid, non-reactive, nanoparticle-modified EP resin (CSR–resin), measuring parameters *A*_T_ = 0.3 K, τ = 60 s, and *q* = ±3*10^–3^ K/s.

Thus, in accordance with this assumption, the quasi-static and dynamic thermal volume expansion coefficients β_stat_ and β′, respectively, almost coincide with each other and reach values typical for liquid and modified EP [β′(*T* > *T*_g_) > 5.5 × 10^–4^ K^–1^; cf. refs (14 and 15)] in the whole temperature range from 10 to 85 °C. The accompanying thermal loss factor β″ slightly increases with the temperature. This effect is assigned to a dynamic shear deformation within the sample, originating at the prism surface and induced by the temperature modulation. Thus, this is an indirect measure of the dynamic shear viscosity of the resin system.^16^

However, as shown in Figure 3, the refractive indices *N*_mean_(*T*) in cooling and heating nearly perfectly coincide with each other. Hence, there is clearly no evidence for a temperature hysteresis or any kind of optical remanence in the nanoparticle-modified, non-reactive resin system. Consequently, the isolated nanoparticles themselves are not the origin of the observed optical remanence nor is it the cross-linked neat polymer network itself (Figure 2). As an important consequence, the origin of the hysteresis behavior is located at the interface between the nanoparticles and the molecular network of the surrounding epoxy matrix. Thus, what might be the physical origin of the observed optical remanence?

To answer this question, TMOR measurements based on the “jump method” were performed.^17^ This approach allows one to switch off temperature-rate-induced kinetics, usually imposed by a temperature rate during the measurements on the thermal volume expansion coefficient, by performing sequentially very small temperature jumps. Once the sample has reached another state of thermal equilibrium, a subsequent isothermal measurement of the thermal volume expansion coefficient is performed.

Figure 4 shows a comparison of the static (jump-method-based) β_stat_(*T*) and the dynamic (*f* = 17 mHz) thermal volume expansion coefficient β′(*T*).

**Figure 4:**
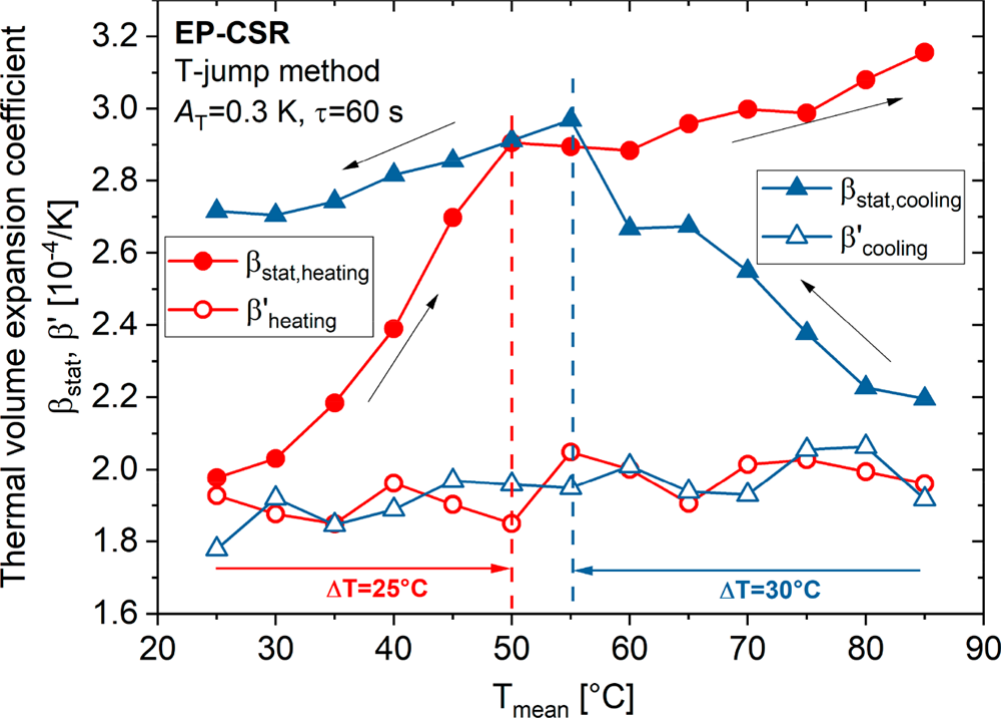
Static and dynamic thermal volume expansion based on the temperature jump method of EP–CSR as a function of the temperature *T*_mean_ (cooling and heating).

In the case of the static thermal volume expansion coefficient β_stat_(*T*), in heating and cooling, a jump is observed. When the heating experiment starts (β_stat,heating_ ∼ 2.0 × 10^–4^ K^–1^; red data set), β_stat_ strongly increases to about 2.9 × 10^–4^ K^–1^ within Δ*T* = 25 °C and flattens out. A similar observation is made when the cooling experiment is performed (blue data set). The thermal volume expansion coefficient β_stat,cooling_ starts at ∼2.2 × 10^–4^ K^–1^, then strongly increases to about 3.0 × 10^–4^ K^–1^ within Δ*T* = 30 °C, and remains on that level. Thus, the temperature-induced jump delays of β_stat_(*T*) are rather similar during heating and cooling. These effects cannot be related to temperature rates, which are omitted using the temperature jump method, but must rather be induced by the temperature difference.

On the other hand, the dynamic thermal volume expansion coefficient β′(*T*) behaves rather inconspicuously (open circles and triangles) and does not show any indication for a pronounced secondary relaxation in the glassy state of the EP–CSR system, which holds to be true during cooling and heating. In contrast to β_stat_(*T*), the data of β′(*T*) does not show any temperature anomaly, and in the margin of error, both curves even coincide and yield absolute β′ values, which slightly increase with the temperature. This also means that the measured β′(*T*) data, at the extremely low temperature modulation frequency of *f* = 17 mHz, have to approximate static thermal volume expansion properties. As a consequence, the static quantity, β_stat_(*T*), cannot represent the true static thermal volume expansion. Otherwise, corresponding β_stat_(*T*) must coincide with the β′(*f* = 17 mHz) curves. Also, it must be concluded that thermal volume expansion is not responsible for the observed optical remanence. The considerable deviations between β_stat_(*T*) and β′(*f* = 17 mHz) must be due to the superposition of an additional physical parameter, beyond pure thermal expansion! Revisiting the Lorenz–Lorentz equation (eq 1), this parameter can be identified as the specific refractivity *r*, representing changes of the optical polarization. The specific refractivity is independent of changes of the electronic dipole density,^18^ i.e., mass density ρ. Usually, *r* is found to be approximately constant if no chemical or physical changes in the bonding situation take place. In such cases, β_stat_(*T*) represents the static thermal volume expansion coefficient. In the current investigation, the difference [β_stat_(*T*) – β′(*T*)] is caused by a temperature variation of *r*, in the glassy state. This finding suggests that molecular bonds close to the interface between the CSR nanoparticles and the epoxy matrix significantly change the strength of their dipole moments, e.g., open or close under the influence of the temperature without changing the anharmonicity of the average molecular interaction potential,^19^ i.e., the thermal volume expansion. Interestingly, this whole process takes place in the glassy state of the nanoparticle-modified epoxy. This means that the glassy state, which is a macroscopic property, does not hinder bond exchange processes, at least at nanoparticle–polymer interfaces (and/or within the interphase). Thus, this also confirms the special role of the optical polarization in the formation of optical remanence in EP–CSR, beyond thermal volume expansion.

In the study presented here, temperature-induced molecular bonding changes in the vicinity of nanoparticles within a polymer network have been investigated. Using temperature-modulated optical refractometry, the thermo-optical and thermomechanical properties during heating and cooling were simultaneously studied. The results demonstrate the existence of temperature-induced refractive index hysteresis (or optical remanence). This phenomenon even persists under quasi-static temperature changes, as shown by the temperature jump method. The simultaneously measured dynamic thermal volume expansion coefficient does not show these hysteresis features and, thus, indicates that the observed optical hysteresis is actually independent of the thermal volume expansion behavior. Therefore, the observed optical remanence is attributed to unexpected changes of the dipole strength of bonds (e.g., by opening and closing of bonds) at the interface between the CSR nanoparticles and the epoxy matrix. With regard to the observed changes of the dipole strength of the nanoparticle-modified EP–CSR, it is at least surprising that the observed bond exchange at the respective interface can take place in the glassy state of the nanocomposite. However, under thermal linear response conditions, this switching of molecular bonds does not affect the thermomechanical properties of the nanocomposite. The observed optical hysteresis anomaly thus has no implications for the mechanical performance of nanoparticle-modified polymer materials.

## Data Availability

The research data underlying this study are openly available in Zenodo (an open repository developed under the European OpenAIRE program) at https://doi.org/10.5281/zenodo.10783429.

## Supporting Information Available

Details on the used materials and sample manufacturing, further details on temperature-modulated optical refractometry, and effect of the temperature rate, aging during the experiments, and statistical validity (PDF)
Transparent Peer Review report available (PDF)
