# Supplementary material for: Temperature-Controlled Molecular Bonding Hysteresis: Interphase Dynamics of a Nanoparticle-Modified Polymer Network
Source: J Phys Chem Lett. 2024 Mar 25;15(13):3576–80. doi: 10.1021/acs.jpclett.4c00406 (PMC11000236; doi:10.1021/acs.jpclett.4c00406)
Supplement: Supplementary file 1 — jz4c00406_si_001.pdf [file jz4c00406_si_001.pdf]

Supporting Information for

Temperature-controlled Molecular Bonding Hysteresis: Interphase Dynamics of a Nanoparticle-modified Polymer Network

Andreas Klingler*, Bernd Wetzel, Jan-Kristian Krüger*

*Corresponding authors. Email: andreas.klingler@ivw.uni-kl.de, jan-kristian.krueger@ivw.uni-kl.de

This PDF file includes:

- Details on the used materials and sample manufacturing
- Further details on Temperature-modulated optical refractometry
- Effect of temperature rate, aging during the experiments and statistical validity

Materials and sample manufacturing:

In the present work a commercially available, nanoparticle modified, bifunctional cycloaliphatic epoxy resin was investigated (KaneAce MX-553, from Kaneka Belgium N.V., Belgium). Its epoxy equivalent weight is given as 192 g/eq., considering the nanoparticles as non-reactive moieties. The masterbatch resin contains 30 ± 1 wt.-% of 100 nm-sized, core-shell structured nanoparticles (CSR-resin). The particle core is made of Polybutadiene (PBd). For orientation, depending on the isomerism, typical glass transition temperatures of PBd are in the range of -107 to -95 °C (Ref.[1]). However, no information are available on the molecular weight of the core or on the shell properties.

As a neat reference, a cycloaliphatic epoxy resin was purchased from Merck KGaA, Germany (3,4-Epoxycyclohexylmethyl-3,4-epoxycyclohexancarboxylat, CAS: 2386-87-0, $m_w = 252\ g/mol$).

For polymerization, the neat resin and the CSR modified resin (MX-553) were respectively mixed with a stoichiometric amount of a cycloaliphatic anhydride (Aradur 917-CH from Huntsman Corp., $m_w = 166\ g/mol$) at 40°C. After 20 minutes 1.8 wt.-% of 1-methylimidazole (1-MI, DY070 from Huntsman Corp., average molecular weight $m_w = 82.1\ g/mol$) was added to the mixtures (with regard to the mass of the epoxy resin) to initiate the cross-linking reaction between the epoxy resins and the anhydride curing agent. Details on the curing reaction between epoxies and anhydride curing agents can be found elsewhere[2,3].

The mixture was then stirred for another 10 minutes and subsequently cast into glass moulds, followed by a three-step curing cycle: (1) 90°C for 4 h, (2) 2 h at 105 °C, and (3) 140 °C for 4 h. After cooling to room temperature, the samples were extracted and machined to cuboid specimens, having dimensions of about 4 x 4 x 3.8 mm^3. The top and bottom sample surfaces were smooth and plain. Thus, no additional surface preparation was performed. 30 wt.-% nanoparticles in the epoxy carrier resin (CSR-resin) corresponds to about 19 vol.-% of nanoparticles in EP-CSR, based on the densities of the constituents (MX553, Aradur 917-CH and 1-MI).

In the main text, *CSR-resin* denominates the non-reactive nanoparticle modified resin (MX553). The cured and CSR particle modified polymer is referred to as *EP-CSR* and the neat and cured cycloaliphatic resin is referred to as *EP-Neat*.

The glass transition temperatures, measured via dynamic mechanical analysis (Q800 from TA Instruments Inc. (USA), single cantilever mode, heating rate of $2\,K/min$, measuring frequency of $f = 10\,Hz$, deformation control: $\varepsilon = 0.041\,\%$, sample dimensions: 35 x 10 x 3.8 mm³) were $T_{g,dyn} = 227°C$ and $T_{g,dyn} = 210°C$ for EP-Neat and EP-CSR, respectively.

Temperature-modulated optical refractometry:

Temperature-modulated optical refractometry (TMOR)[4,5] (or Thermo-optical oscillating refraction characterization (TORC), by *Anton Paar OptoTec GmbH*, Germany) measures optically, via a modified Abbe refractometer, the complex refractive index n_ω^* of samples, from the liquid to the solid state, and thus provides access to the static and dynamic thermal volume expansion coefficients, β and β^*, respectively.

The very basis is the Lorenz-Lorentz relationship[6–8]:

$$\frac{n(T)^2-1}{n(T)^2+2} = r \cdot \rho(T) \qquad \text{[Eq. 1]}$$

where n is the refractive index, r the specific refractivity and ρ the mass density.

The average magnitude of the electronic dipole moments is reflected by *r*, the mass density ρ is proportional to the electronic dipole density of the sample. Hence, temperature-induced changes of n are caused by a modification of the electronic dipole system, when the material shrinks or expands.

If the refractive index is a function of the temperature, i.e. $n = n(T)$, two non-correlated coefficients, $\beta(T)$ and $\psi(T)$, can be derived:

$$\frac{-6n(\mathrm{T})}{(\mathrm{n}^2(\mathrm{T})-1)(\mathrm{n}^2(\mathrm{T})+2)}\frac{dn(\mathrm{T})}{dT} = \psi(T) + \beta(T) \qquad \text{[Eq. 2]}$$

with

$\beta(T) = \frac{-1}{\rho}\frac{d\rho}{dT}$ and $\psi(T) = -\frac{1}{r}\frac{dr}{dT}$.

When the refractive index is modified by changes of the temperature T, β is called the "thermal volume expansion coefficient" and ψ is called the specific refractivity[8]. If temperature-induced changes of ψ are very small in comparison to those of β, i.e. $\psi \ll$

β, the following simple relation between the thermo-optical coefficient dn/dT and the static thermal volume expansion coefficient β is obtained:

$$\beta = \frac{-6n(T)}{(\mathrm{n}^2(\mathrm{T})-1)(\mathrm{n}^2(\mathrm{T})+2)} \frac{dn(T)}{dT} \qquad \text{[Eq. 3]}$$

In the case of TMOR, an average temperature signal T_{iso} is superimposed with a small sinusoidal temperature perturbation T_{mod} (satisfying linear response theory):

$$T(t) = T_{\mathrm{iso}} + T_{\mathrm{mod}} = T_{\mathrm{iso}} + A_T \sin(2\pi f t) \qquad \text{[Eq. 4]}$$

where A_T is the amplitude of the temperature perturbation and f the temperature modulation frequency. Then, the complex thermo-optical coefficient (TOC) is obtained:

$$\Psi^* = \frac{\partial n_\omega^*}{\partial T} = \frac{A_n}{A_T} \mathrm{e}^{\mathrm{i}\Phi} \qquad \text{[Eq. 5]}$$

where n_ω^* is the frequency-dependent, complex refractive index response of the material to the temperature perturbation, A_T is the amplitude of the excitation temperature, A_n is the amplitude of that refractive index response and ϕ is the phase lag between the sinusoidal temperature perturbation and the refractive index response. Making use of the Lorentz-Lorenz equation (Eq.1), the TOC (Eq. 5]) can be separated into a real and an imaginary part of the dynamic thermal volume expansion coefficient $\beta^*(t,T,f) = \beta'(t,T,f) + i\beta''(t,T,f)$:

$$\beta'(t,T,f) = \frac{-6(N_{\mathrm{mean}}(t,T))}{[N_{\mathrm{mean}}^2(t,T)-1][N_{\mathrm{mean}}^2(t,T)+2]} \frac{A_n(t,T)}{A_T} cos\big(\Phi(t,T,f)\big), \qquad \text{[Eq. 6]}$$

$$\beta''(t,T,f) = \frac{-6(N_{\mathrm{mean}}(t,T))}{[N_{\mathrm{mean}}^2(t,T)-1][N_{\mathrm{mean}}^2(t,T)+2]} \frac{A_n(t,T)}{A_T} sin\big(\Phi(t,T,f)\big). \qquad \text{[Eq. 7]}$$

where N_{mean} is the average refractive index response over one modulation period.

A detailed description of the TMOR theory can be found elsewhere (cf. Ref.[4]).

TMOR offers an outstanding relative and absolute accuracy of $\Delta n = 10^{-6}$ and 10^{-5}, respectively. The accuracy of the temperature controller is $\Delta T = \pm 0.03\ K$.

In the present study, the measurements were performed using a TORC 5000 (*Anton Paar OptoTec GmbH*, Germany). The experimental investigation of the liquid system (CSR-resin) and the cross-linked systems (EP-CSR and EP-Neat) were performed in a temperature range from 25°C to 85°C using temperature rates in between $3 \cdot 10^{-3} K/s$ to $10^{-4} K/s$. Prior to the measurements, the system was calibrated against

deionized water ($n_D = 1.332986$ at $T = 20\ °C$, H_2O, 18.02 g/mol, from Carl Roth GmbH + Co. KG, Germany).

Effect of temperature rate, aging during the experiments and statistical validity:

Figure S1 shows the hysteretic refractive index response of EP-CSR of the same sample, based on three different experiments, performed one after the other. The hysteresis behaviour persists independent of the temperature rate. Even when kinetic influences are completely switched off, using the temperature jump method, the refractive index behaves hysteretic. This re-emphasizes that the nanoparticle-induced hysteresis behaviour is not kinetically induced.

Even more, considering the time of the experiments (e.g. using solely a temperature rate of $0.0001K/s$ takes about 2 weeks time to complete a heating-cooling-heating cycle), it can be stated that the observed hysteresis does not underlie any physical and/or chemical aging effect.

Since the experimental conditions do not affect the phenomenon, the data sets nicely illustrate the high precision reproducibility of the measurements.

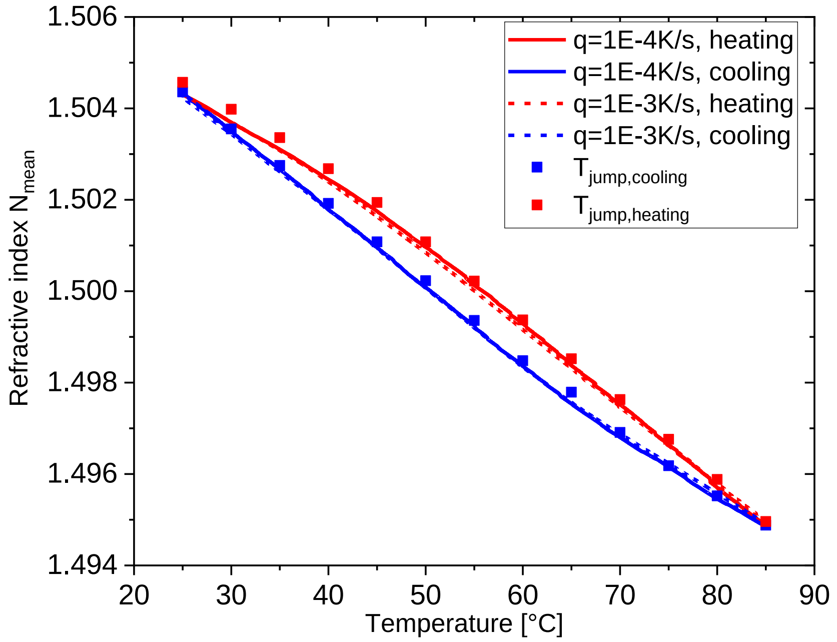

Figure S1: Effect of temperature rate on the refractive index response

References:

(1) Salamone, J. C. *Polymeric Materials Encyclopedia*; CRC Press: Boca Raton, 1996.
(2) Woo, E. M.; Seferis, J. C. Cure Kinetics of Epoxy/Anhydride Thermosetting Matrix Systems. *Journal of Applied Polymer Science* **1990**, *40* (7–8), 1237–1256. https://doi.org/10.1002/app.1990.070400713.
(3) Matějka, L.; Lövy, J.; Pokorný, S.; Bouchal, K.; Dušek, K. Curing Epoxy Resins with Anhydrides. Model Reactions and Reaction Mechanism. *Journal of Polymer Science: Polymer Chemistry Edition* **1983**, *21* (10), 2873–2885. https://doi.org/10.1002/pol.1983.170211003.
(4) Müller, U.; Philipp, M.; Thomassey, M.; Sanctuary, R.; Krüger, J. K. Temperature Modulated Optical Refractometry: A Quasi-Isothermal Method to Determine the Dynamic Volume Expansion Coefficient. *Thermochimica Acta* **2013**, *555*, 17–22. https://doi.org/10.1016/j.tca.2012.12.011.
(5) Müller, U.; Krüger, J. K. Temperature Modulated Refractive Index Measurement. patent no. 2609417, July 3, 2013. https://patentscope.wipo.int/search/en/detail.jsf?docId=EP82248870.
(6) Lorentz, H. A. Ueber die Beziehung zwischen der Fortpflanzungsgeschwindigkeit des Lichtes und der Körperdichte. *Ann. Phys. Chem.* **1880**, *245* (4), 641–665. https://doi.org/10.1002/andp.18802450406.
(7) Lorenz, L. Ueber die Refractionsconstante. *Ann. Phys. Chem.* **1880**, *247* (9), 70–103. https://doi.org/10.1002/andp.18802470905.
(8) Böttcher, C. J. F. *Theory of Electric Polarisation*; Elsevier Publishing Company, 1952.
