# Supplementary material for: Temperature-Controlled Molecular Bonding Hysteresis: Interphase Dynamics of a Nanoparticle-Modified Polymer Network
Source: J Phys Chem Lett. 2024 Mar 25;15(13):3576–80. doi: 10.1021/acs.jpclett.4c00406 (PMC11000236; doi:10.1021/acs.jpclett.4c00406)
Supplement: Supplementary file 2 — jz4c00406_si_002.pdf [file jz4c00406_si_002.pdf]

jz-2024-00406c.R1

Name: Peer Review Information for "Temperature-controlled Molecular Bonding Hysteresis: Interphase Dynamics of a Nanoparticle-modified Polymer Network"

First Round of Reviewer Comments

Reviewer: 1

Comments to the Author

This manuscript reports a temperature-induced refractive index hysteresis due to changes in dipole strength of bonds close to the interfaces between CSR nanoparticles and epoxy matrix. The temperature-dependent refractive index and thermal expansion coefficient were measured using a novel temperature-modulated optical refractometry (TMOR). The difference in the static and dynamic thermal volume expansion coefficients indicates a temperature-dependent specific refractivity, due to the changes in the molecular bonds close to the interface near the glassy state. This is an interesting phenomenon which has not been reported in previous work. The most significant part of this work is the probe of the interphase dynamics at the nanoparticle/polymer interfaces, which has a strong implication in designing nanocomposites with excellent mechanical properties. The manuscript is well written, and I recommend the publication of this work in JPCL after addressing the following minor issues.

1. In Fig. 2, the refractive index of epoxy during cooling is shown. How about any hysteresis observed for the neat epoxy?

2. Page 8, line 48-50: Regarding the bonds open or close under the influence of temperature, could the authors provide details of the possible reaction that occurred at the interface?

3. Fig. 4: could the authors comment on the data scattering of measured thermal volume expansion coefficients (e.g., error bars of the data)?

Reviewer: 2

Comments to the Author

This contribution provides some highly interesting data on interphases in epoxy nanocomposites (Fig.1).

Unfortunately, the (statistical) validation and the rigour of the analysis and argumentation show considerable deficits.

Therefore, this paper may be publishable, but major revision is needed. I would like to be invited to review any future revision.

Comments/issues:

(1)

p.2/l.34: It is not the "shell" that forms the interphase, but the interaction of the cross-linking epoxy with the shell.

What type of interactions are expected there? Interpenetration network? Chemical adhesion? ...

(2)

p.2/l.53: 30 wt.-% is not interesting. What is the vol-%? Knowing the number of particles per volume of composite is

important to estimate the interphase volume fraction and, thereby, if it at all is significant for the overall response.

100nm is a particle size for which the famous size effect may already be neglible, depending on the interphase thickness.

(3)

p2./l.58: How thick is the shell, and what is it made of?

(4)

Fig.1: Fantastic and very interesting data! But: Is this a single measurement?

What happens when the same specimen is tested for a second time, maybe a few days later?

Is the composite stable and fully cured? Or might the hysteresis be caused by physical or chemical ageing?

A decent characterization of the cured composite is required.

(5)

p.3/l.37: should be 25 C, not 20 C

(6)

In Eq.1, r is of dimension cm /g, inverse to mass density rho. On p.4/l.47, rho becomes the dipole number density.

Outline the correlation of rho to the dipole density.

What dipoles do the authors refer to? The Lorentz-Lorenz-eq. only holds for dipole-free media where the light induces dipoles.

The presence of permanent dipoles complicates the situation considerably:

(i) They modify the polarization state of the molecules and, therefore, the polarization

that the light can induce on top.

(ii) They modify the local electric field strength of the light as the stimulus for induced polarization.

(7)

Fig.2: legend is incomplete (N_mean is missing), and different line styles for N_mean and beta_stat would be better

I like the idea of a step-by-step elimination of possible root causes for the N_mean hysteresis, i.e. by comparing the

behaviour of the components. But this requires identical and stable experimental conditions:

(8)

Heating and cooling rates for EP-neat (fig.2) & CSR-resin (fig.3) are 20 and 10 times larger than for EP-CSR in Fig.1.

This at least deserves a comment on comparability of the experiments. Furthermore, fig.2 only shows cooling, which is

not acceptable regarding a comparison. These inconsistencies make the whole argumentation highly vulnerable.

(9)

Fig.4: Why do the beta_stat curves for cooling and heating start and end at different values?

Shouldn't these values coincide? Was this not a continuous experiment, first heating, then cooling, or vice versa?

Could this be caused by any kind of ageing during the experiment? In this case, Fig.1 would just show an artifact.

(10)

Fig.1: What is the meaning of T_kink=68 C? That is not mentioned in the text.

(11)

The refractive index hysteresis in EP-CSR (Fig.1) could also be a consequence of local residual mechanical strains

from hindered curing shrinkage of the epoxy at the particle surfaces. It is well known that the refractive index in

epoxies changes due to mechanical strains, which would here be overlaid by thermal expansion.

Such shrinkage stresses cannot have relaxed away here since all experiments were conducted deep in the glassy state

of the composite. This would offer a different and (admittedly) less spectacular hypothesis than drawn in the paper:

"...unexpected changes of the dipole strength of bonds (e.g. by opening and closing of bonds) close to the interface,

between the CSR nanoparticles and the epoxy matrix."

Which type of bonds would be opened and closed? These conclusions are rather vague and not well supported by the data presented.

Comments on the "Supporting Information":

(1)

What are chemical reactions between the three components?

(2)

After the three curing steps: How complete is the curing reaction?

(3)

4x4x3.8 mm is not a "cubic" specimen

(4)

DMA mode? Tension, compression, bending...

(5)

Eq.4: How are N_mean and n from Eq.3 related?

(6)

Eq.4/5: Is capital Phi the same function as lower-case phi in Eq.2?

Author's Response to Peer Review Comments:

Letter to the editor and the reviewers:

Manuscript title:
Temperature-controlled Molecular Bonding Hysteresis: Interphase Dynamics of a Nanoparticle-modified Polymer Network

Dear Editor,
dear Reviewers,

first of all, thank you very much for your time and your valuable comments to this manuscript! We have addressed all of your comments in detail below, and partially modified the manuscript and the supporting information, respectively (highlighted in yellow).

Reviewer #1:
This manuscript reports a temperature-induced refractive index hysteresis due to changes in dipole strength of bonds close to the interfaces between CSR nanoparticles and epoxy matrix. The temperature-dependent refractive index and thermal expansion coefficient were measured using a novel temperature-modulated optical refractometry (TMOR). The difference in the static and dynamic thermal volume expansion coefficients indicates a temperature-dependent specific refractivity, due to the changes in the molecular bonds close to the interface near the glassy state. This is an interesting phenomenon which has not been reported in previous work. The most significant part of this work is the probe of the interphase dynamics at the nanoparticle/polymer interfaces, which has a strong implication in designing nanocomposites with excellent mechanical properties. The manuscript is well written, and I recommend the publication of this work in JPCL after addressing the following minor issues.

Answer: Thank you very much for your initial comment. We think, this is a nice summary of what we intended to convey.

1. In Fig. 2, the refractive index of epoxy during cooling is shown. How about any hysteresis observed for the neat epoxy?

   Answer: *In the case of the neat epoxy, we did not observe any kind of optical hysteresis behaviour. This can also be seen from the figure. Even though only the cooling part of EP-Neat is shown, the measured data shows a quasi-perfect linear relationship between the refractive index N_{mean} and the temperature T, representing equilibrium conditions of EP-Neat in this temperature interval.*
   We added this to the text, just above Figure 2.

2. Page 8, line 48-50: Regarding the bonds open or close under the influence of temperature, could the authors provide details of the possible reaction that occurred at the interface?

   Answer: *Unfortunately, we do not have any information on the chemical/physical interactions between the nanoparticles and the resin/hardener mixture. The material is commercially available and a classical cycloaliphatic epoxy resin. The nanoparticles have been synthesised via an emulsion polymerization process and are in a range of 100 nm, according to the manufacturer. That has been confirmed using SEM analysis, see below. The figure shows a fracture surface of EP-CSR. The dark*

"holes" represent fracture features that have been created by the particles and can usually be used to get an idea at least of the particle diameter. Even more, the particles are evenly distributed. This means, we are here indeed dealing with an optically homogenous material.

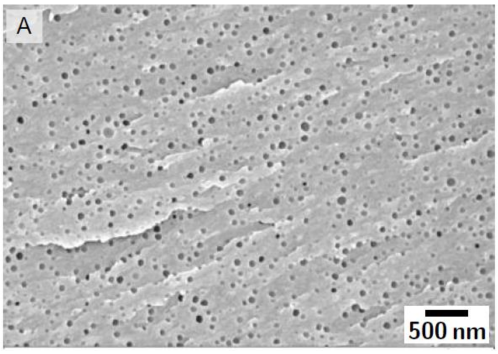

3. Fig. 4: could the authors comment on the data scattering of measured thermal volume expansion coefficients (e.g., error bars of the data)?

Answer: *The relative and absolute accuracy of the refractive index measurement via TMOR is* $\Delta n = 10^{-6}$ *and* 10^{-5}*, respectively. The accuracy of the temperature controller is* $\Delta T = \pm 0.03\ K$*. The information is provided to the reader in the supporting information. Nevertheless, to give you an idea of the accuracy please see Figure 3 in the manuscript. Figure 3 shows a repetition of the EP-resin measurements, once in heating, once in cooling. The measurements are that precise that you cannot even see any data ripple or data scatter. The small shift of* N_{mean} *(the minor difference between the dashed and the bold line) is rather of physical origin.*

Also, please see the figure below, which shows a cooling-heating-cooling run of the neat resin (without nanoparticles). Here, the data nearly perfectly overlaps, since the low molecular weight resin is at all times in an equilibrium state.

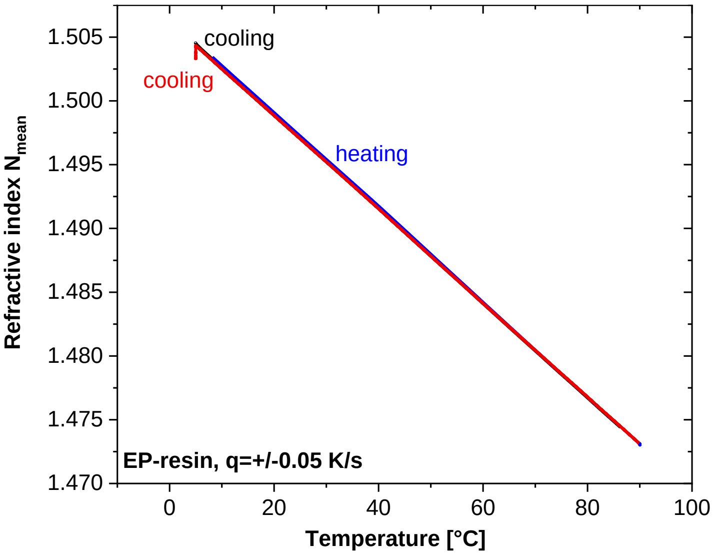

Reviewer #2:

This contribution provides some highly interesting data on interphases in epoxy nanocomposites (Fig.1). Unfortunately, the (statistical) validation and the rigour of the analysis and argumentation show considerable deficits.
Therefore, this paper may be publishable, but major revision is needed. I would like to be invited to review any future revision.

Answer: *Dear reviewer, thank you very much for your review. As a preliminary comment, the manuscript has been created based on data, whose original intention was not to investigate the interphase dynamics of nanoparticle-modified polymer networks. We rather stumbled over this finding in our data and were excited about it. Thus, the data sets are not as systematic as one could expect from a study that has rigorously been planned. However, this does not affect the accuracy or the statistical validity of the measurements. Please find below our answers to your questions and some additional information with regard to the statistical validity of the data.*
We will make the research data available in an open data repository to ensure the greatest possible transparency (see data availability statement after the conclusion section in the manuscript) and we will also participate in the transparent peer review process JPCL offers.

From our perspective, the experimental findings and the physical insights are an utmost interesting starting point for future research projects/ideas in the field of polymer interphases and molecular properties. This was also one of the reasons why we chose to submit the work in the letter format to "The Journal of Physical Chemistry Letters".

1. p.2/l.34: It is not the "shell" that forms the interphase, but the interaction of the cross-linking epoxy with the shell. What type of interactions are expected there? Interpenetration network? Chemical adhesion? ...

   Answer: *You are correct. It is not the nanoparticle shell that forms the interphase but the interaction between the nanoparticle shell and the surrounding polymer matrix. We modified the respective section.*

   With regard to your second comment, we can only speculate about the interaction between the nanoparticles and the resin. That is where we are a little bit blindfolded. For example, based on the SEM investigations shown in the answer to the second question of reviewer #1, we presume that the particles interact dominantly physically with the polymer matrix.

2. p.2/l.53: 30 wt.-% is not interesting. What is the vol-%? Knowing the number of particles per volume of composite is important to estimate the interphase volume fraction and, thereby, if it at all is significant for the overall response. 100nm is a particle size for which the famous size effect may already be neglible, depending on the interphase thickness.

   Answer: *That is also correct, and we adapted this comment in the manuscript as well as in the supporting information: 30 wt.-% nanoparticles in the epoxy carrier resin corresponds to about 19 vol.-% of nanoparticles in EP-CSR, based on the densities and the EP constituents (resin with particles, hardener and accelerator).*

With regard to your comment on the nanoparticle size effect: investigating the effect of the particle size, the particle concentration or other parameters would indeed be highly interesting, but it was not the aim of the present manuscript. If the nano size is indeed the driving parameter in this investigation is open for further discussion.

3. p2./l.58: How thick is the shell, and what is it made of?

   Answer: *The CSR-resin is a commercially available product and only little information is available on the nanoparticle composition. We presume that the nanoparticles have a shell thickness of a few nanometers, thus a few molecular units. For now, unfortunately, we do not have further information on the nanoparticles, besides the ones provided in the supplementary information.*

4. Fig.1: Fantastic and very interesting data! But: Is this a single measurement? What happens when the same specimen is tested for a second time, maybe a few days later? Is the composite stable and fully cured? Or might the hysteresis be caused by physical or chemical ageing? A decent characterization of the cured composite is required.

   Answer: *The data shown in Figure 1 is a single measurement, performed in a consecutive heating and cooling run, using a temperature rate of $10^{-4} K/s$ (in heating and cooling). The same sample has subsequently been used for another temperature rate experiment using a temperature rate of $10^{-3} K/s$. The results are shown below. As can be seen, the hysteretic behaviour is persistent and both measurements overlap; even more, they close again at the same temperature. Hence, the temperature-rate does not affect the data set. This holds to be true at least in the investigated temperature-rate range and is confirmed by the data obtained from the temperature jump method (squared points). This also means, even though the material underwent several, really long heating and cooling cycles (~weeks), the morphology of the material did not change. To say it in advance with regard to your comment #8, the figure below demonstrates that the measuring conditions, i.e. the temperature-rates can be considered as quasi-static conditions.*

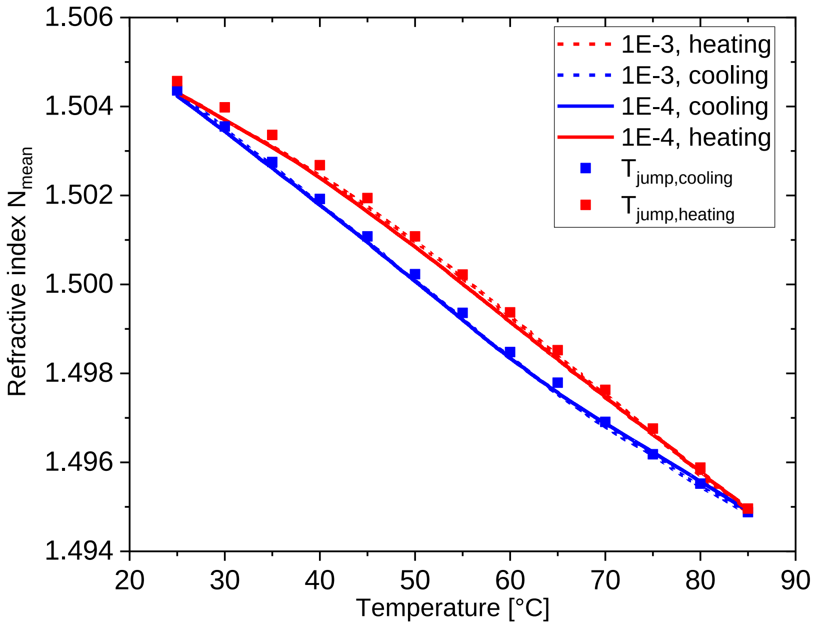

Addressing your comment about the effect of physical and chemical aging in more detail: EP-CSR and EP-Neat are both based on the same initial components (base resin, hardener, accelerator), cured in the same way and show a glass transition temperature of about 210°C and 227°C, respectively. The figure above shows the hysteretic refractive index response of EP-CSR based on three different experiments, performed one after the other of the same sample. Now considering the time of the experiments (e.g. using solely a temperature rate of $0.0001K/s$ *takes about 2 weeks time to complete a heating-cooling cycle), it can be stated that the observed hysteresis does not underlie any physical and/or chemical aging effects.*

If and how aging might affect the hysteresis behaviour at other experimental boundary conditions (e.g. at higher temperatures) is an additional question that might indeed be of interest for future works. Staying far below any kind of additional temperature-induced molecular activities (post-curing, dynamic exchange, molecular motions) and especially far from the glass transition, we consider EP-Neat and EP-CSR as stable (see again the figure above, where the same sample has been heated and cooled for very long time scales).

We have added the above figure to the supporting information, to supplement the data sets with regard to a statistical validation, as well as to demonstrate the similarity of the measuring conditions.

5. p.3/l.37: should be 25°C, not 20°C

   Answer: *Thank you, we corrected it.*

6. In Eq.1, r is of dimension cm^3/g, inverse to mass density rho. On p.4/l.47, rho becomes the dipole number density. Outline the correlation of rho to the dipole density. What dipoles do the authors refer to? The Lorentz-Lorenz-eq. only holds for dipole-free media where the light induces dipoles. The presence of permanent dipoles complicates the situation considerably:
   (i) They modify the polarization state of the molecules and, therefore, the polarization that the light can induce on top.
   (ii) They modify the local electric field strength of the light as the stimulus for induced polarization.

   Answer: First, it is important to point out that we are only dealing with dipoles that are induced at optical frequencies (THz region). At these frequencies of the electromagnetic field all other types of dipoles (e.g. permanent dipoles) are clamped. Thus, we are only talking about the ***electric polarization****.*
   Secondly, the refractive index is related to the dielectric constant via $n = \sqrt{\varepsilon_\infty}$*. The polarization, which is the number of electric dipoles per volume and thus represents the dipole number density, can then be expressed as a function of the mass density* ρ*. In the end* ρ *is proportional to the dipole number density.*

   We modified this section in the manuscript to account for the proportionality, since it indeed is not the same as the mass density! Thank you for this hint.

7. Fig.2: legend is incomplete (N_mean is missing), and different line styles for N_mean and beta_stat would be better

Answer: *We modified the line styles and completed the legend.*

8. I like the idea of a step-by-step elimination of possible root causes for the N_mean hysteresis, i.e. by comparing the behaviour of the components. But this requires identical and stable experimental conditions:

   Heating and cooling rates for EP-neat (fig.2) & CSR-resin (fig.3) are 20 and 10 times larger than for EP-CSR in Fig.1. This at least deserves a comment on comparability of the experiments. Furthermore, fig.2 only shows cooling, which is not acceptable regarding a comparison. These inconsistencies make the whole argumentation highly vulnerable.

   Answer: *As we stated in our initial comment to this review, the data sets are not as systematic as they could have been, if the study would have been a planned action. Here, it was much more a scientific finding that we think needs to be shared with the community, while still keeping the data-based physics on the highest level. This means the temperature rates we are dealing with are ultra slow and can even be considered as quasi-static. Indeed, it would enhance the appearance of the manuscript if the data would have been measured with the same temperature rates, but re-measuring a complete heating/cooling cycle with a temperature rate of* $0.0001K/s$ *over a temperature interval from 25°C to 85°C and back to 25°C would take about two weeks. As has been shown in the figure to your comment #4, the data sets, even though measured with different temperature rates (*10^{-4}*K/s and* 10^{-3}*K/s), nearly perfectly match. We added a statement about the similarity of the measurement conditions to the supplementary information.*

   With regard to your statement about Figure 2: EP-Neat does not show any optical hysteresis behaviour, which can be seen from the quasi-perfect linear relationship between the refractive index N_{mean} *and the temperature T, representing equilibrium conditions of EP-Neat in this temperature interval. Hence, the additional measurement would not help the physics of the phenomenon. We added this to the text, just above Figure 2 and added a straight line fit to the data set. We also commented on it in the subtitle of the figure.*

9. Fig.4: Why do the beta_stat curves for cooling and heating start and end at different values? Shouldn't these values coincide? Was this not a continuous experiment, first heating, then cooling, or vice versa? Could this be caused by any kind of ageing during the experiment? In this case, Fig.1 would just show an artifact.

   Answer: *The experiment was first done in heating and then in cooling. This means, the temperature jumps were performed consecutively starting at 25°C up to 85°C, and then consecutively down again to 25°C. In between each temperature jump, the temperature was kept constant for about 2 hours. During that time, the sample had time to reach an equilibrium state.*
   The reason that the beta_stat curves do not coincide at 25°C and 85°C is more a mathematical one. Beta_stat does not show the real static thermal expansion value, as stated in the middle of page 8! Beta_stat is given as

$$\beta_{stat} = \frac{-6n(T)}{(\mathrm{n}^2(\mathrm{T})-1)(\mathrm{n}^2(\mathrm{T})+2)}\frac{dn(T)}{dT}$$

Thus, it is proportional to $dn(T)/dT$. Hence, in the present case, the reason for the difference between "beta_stat" in cooling and heating at e.g. 85°C (which is just a pseudo value, since it biased by the specific refractivity, $r \neq const.$) is that the refractive index, due to temperature-induced hysteresis effect, has different slopes in heating and in cooling.
Please note that we added the mathematical background of Beta_stat to the TMOR description in the supporting information.

10. Fig.1: What is the meaning of T_kink=68°C? That is not mentioned in the text.

    Answer: *We removed T_kink from the figure. It was originally thought to emphasize an aspect of the figure, but we omitted this.*

11. The refractive index hysteresis in EP-CSR (Fig.1) could also be a consequence of local residual mechanical strains from hindered curing shrinkage of the epoxy at the particle surfaces. It is well known that the refractive index in epoxies changes due to mechanical strains, which would here be overlaid by thermal expansion. Such shrinkage stresses cannot have relaxed away here since all experiments were conducted deep in the glassy state of the composite. This would offer a different and (admittedly) less spectacular hypothesis than drawn in the paper: "...unexpected changes of the dipole strength of bonds (e.g. by opening and closing of bonds) close to the interface, between the CSR nanoparticles and the epoxy matrix." Which type of bonds would be opened and closed? These conclusions are rather vague and not well supported by the data presented.

    Answer: *We thought about your argument, but if mechanical stresses would be imparted into the network, e.g. at the nanoparticle interphases, it would not cause a hysteresis effect. The heating and cooling behaviour of the refractive index would be similar "in the same direction" during heating and cooling, since stresses would relax when the material is heated and become active again when the material is cooled (not considering a possible entropy-elastic response). Hence, the refractive index would eventually indicate such a behaviour but the data sets would overlay each other during heating and cooling and not show a hysteresis effect.*

    With regard to the bond opening and closing: we do not know. Based on the data we report in this manuscript, we can say that something is happening at the particle/matrix interphase, but further research would be required to pin point e.g. the involved type of bonds.

<u>Comments on the "Supporting Information":</u>

1. What are chemical reactions between the three components?

    Answer: *The curing reaction between epoxies and anhydride curing agents is rather complex, since several curing reactions can take place and a ring opening reaction can either start at the epoxide group of the epoxy or at the anhydride ring. Also, without an accelerator catalyst the reactivity between an epoxy and an anhydride is quite low. Details on the curing reaction are given e.g. in the following papers:*

    Matějka, L., J. Lövy, S. Pokorný, K. Bouchal, und K. Dušek. „Curing Epoxy Resins with Anhydrides. Model Reactions and Reaction Mechanism". Journal of Polymer Science: Polymer Chemistry Edition 21, Nr. 10 (1983): 2873–85. https://doi.org/10.1002/pol.1983.170211003

Woo, E. M., und J. C. Seferis. „Cure Kinetics of Epoxy/Anhydride Thermosetting Matrix Systems". Journal of Applied Polymer Science 40, Nr. 7–8 (1990): 1237–56. https://doi.org/10.1002/app.1990.070400713

We added the references to the supplementary information.

2. After the three curing steps: How complete is the curing reaction?

   Answer: *The degree of post-curing of the neat polymer system (i.e.* ***EP-Neat****) was found to be in the range of 6%. The degree of post-curing of* ***EP-CSR*** *is unknown. Both materials have been prepared the same way and cured the same way. Also, the post-curing effect of EP-Neat starts beyond 120°C. If the hysteresis behaviour of EP-CSR would have been related to a type of post-polymerization process in the investigated temperature-interval, it would diminish over time, which it does not, as shown in the figure to your question #4. If unreacted groups would be responsible for the hysteresis behaviour, they should also become active in EP-Neat and distort the refractive index (since the materials are comprised of the same reactive components and cured the same way), which they do not.*

3. 4x4x3.8 mm⍰ is not a "cubic" specimen

   Answer: *We changed "cubic" to "cuboid".*

4. DMA mode? Tension, compression, bending...

   Answer: *The DMA experiments were performed in a single cantilever beam setup. The information is given in the supporting information. We highlighted it in green.*

5. Eq.4: How are N_mean and n from Eq.3 related?

   Answer: *n(T) in Eq. 3 refers generally to the refractive index.* N_{mean} *on the other hand is the average refractive index response over one sinusoidal modulation period during the TMOR measurement. We added a comment below the equations.*

6. Eq.4/5: Is capital Phi the same function as lower-case phi in Eq.2?

   Answer: *Yes, it is the phase angle between the temperature excitation and the refractive index response. We changed the lower-case phis in Eq. 4 and 5 to a capital Phi, as shown in Eq. 2.*

jz-2024-00406c.R2

Name: Peer Review Information for "Temperature-controlled Molecular Bonding Hysteresis: Interphase Dynamics of a Nanoparticle-modified Polymer Network"

Second Round of Reviewer Comments

Reviewer: 2

Comments to the Author

The authors have adequately addressed all my questions and comments and I now recommend publication of the manuscript in its current form.

Author's Response to Peer Review Comments:

Dear Editor,

Dear Sir or Madam,

We adapted the requested non-scientific changes to the manuscript and are ready to publish with JPCL.

With kind regards,

Dr. Andreas Klingler
